# Unsupported off-label chemotherapy in metastatic colon cancer

**DOI:** 10.1186/1472-6963-12-481

**Published:** 2012-12-29

**Authors:** Jonas A de Souza, Blase Polite, Monica Perkins, Neal J Meropol, Mark J Ratain, Lee N Newcomer, G Caleb Alexander

**Affiliations:** 1 Section of Hematology/Oncology, The University of Chicago Medicine, Chicago, IL, USA; 2 Comprehensive Cancer Center, The University of Chicago Medicine, Chicago, IL, USA; 3 Center for Interdisciplinary Health Disparities Research, The University of Chicago Medicine, 5841 South Maryland Avenue, MC 2115, Chicago, IL, 60637-1470, USA; 4 UnitedHealthcare, Edina, Minnesota, USA; 5 Division of Hematology and Oncology, University Hospitals Case Medical Center Seidman Cancer Center, Case Western Reserve University, Case Comprehensive Cancer Center, Cleveland, Ohio, USA; 6 Committee on Clinical Pharmacology and Pharmacogenomics and Center for Personalized Therapeutics, The University of Chicago, Chicago, IL, USA; 7 Section of General Medicine and Center for Health and the Social Sciences, University of Chicago, Chicago, IL, USA

**Keywords:** Colorectal cancer, Off-label, Evidence-based medicine, Physician practice patterns

## Abstract

**Background:**

Newer systemic therapies have the potential to decrease morbidity and mortality from metastatic colorectal cancer, yet such therapies are costly and have side effects. Little is known about their non-evidence-based use.

**Methods:**

We conducted a retrospective cohort study using commercial insurance claims from UnitedHealthcare, and identified incident cases of metastatic colon cancer (mCC) from July 2007 through April 2010. We evaluated the use of three regimens with recommendations against their use in the National Comprehensive Cancer Center Network Guidelines, a commonly used standard of care: 1) bevacizumab beyond progression; 2) single agent capecitabine as a salvage therapy after failure on a fluoropyridimidine-containing regimen; 3) panitumumab or cetuximab after progression on a prior epidermal growth factor receptor antibody. We performed sensitivity analyses of key assumptions regarding cohort selection. Costs from a payer perspective were estimated using the average sales price for the entire duration and based on the number of claims.

**Results:**

A total of 7642 patients with incident colon cancer were identified, of which 1041 (14%) had mCC. Of those, 139 (13%) potentially received at least one of the three unsupported off-label (UOL) therapies; capecitabine was administered to 121 patients and 49 (40%) likely received it outside of clinical guidelines, at an estimated cost of $718,000 for 218 claims. Thirty-eight patients received panitumumab and six patients (16%) received it after being on cetuximab at least two months, at an estimated cost of $69,500 for 19 claims. Bevacizumab was administered to 884 patients. Of those, 90 (10%) patients received it outside of clinical guidelines, at an estimated costs of $1.34 million for 636 claims.

**Conclusions:**

In a large privately insured mCC cohort, a substantial number of patients potentially received UOL treatment. The economic costs and treatment toxicities of these therapies warrant increased efforts to stem their use in settings lacking sufficient scientific evidence.

## Background

During the past two decades there have been many advances in the treatment of metastatic colorectal cancer. For half a century, 5-fluorouracil was the only treatment that demonstrated benefit and was thus used as a standard agent. Since the United States Food and Drug Administration (FDA) approved irinotecan, three other cytotoxic agents and three biologic agents have been added to the armamentarium for the treatment of metastatic colorectal cancer. These therapeutic innovations have resulted in an increase in median survival from a baseline of 4 to 6 months (i.e., with supportive care alone), to approximately one year with 5-fluorouracil, and to more than 20 months with the use of sequential chemotherapy including cytotoxic agents and biologics [1].

This scientific progress, associated with the increase in the number of available regimens, has come at considerable cost. The improvement in survival has been accompanied by an estimated 340-fold increase in the cost of drugs for treating colorectal cancer [2,3]. At the same time, although the FDA approves therapies for specific clinical, or labeled, indications, physicians are free to use licensed medicines for both FDA-approved and non-approved, or off-label, uses [4,5]. In oncology, an estimated 50% of prescribed therapies are used off-label [6]. While many of these uses have clinical evidence, and indeed, may reflect the standard of care, there are also off-label uses not supported by evidence, or unsupported off-label (UOL) use.

Given the increased number of regimens and available combinations for metastatic colorectal cancer, coupled with the terminal implications of a diagnosis of metastatic disease, we hypothesized UOL use of chemotherapy commonly occurs in this setting. Our aim was to describe how often UOL chemotherapy occurs in this cohort of patients with metastatic colon cancer.

## Methods

### Data source

We identified patients with colon cancer from the United Healthcare health insurance database, which represents over 70 million privately insured patients across all 50 states, the District of Columbia, and the U.S. Virgin Islands. The database, which captures care delivered by over 700,000 physicians and other health care providers within 5,200 hospitals, contains information on member demographics, and utilization, charges and allowed payments for covered services, including hospitalizations, outpatient procedures, physicians’ office visits and outpatient prescriptions. Data available for each facility and professional service claim include dates of service and International Classification of Diseases (ICD-9-CM) diagnosis codes. Professional service claims also include Level I Healthcare Common Procedure Coding System (HCPCS) codes, also known as Current Procedural Terminology (CPT) procedure codes, as well as Level II HCPCS codes. We defined chemotherapies using codes from the CPT and Level II HCPCS. These claims also provide additional information about the medications dispensed including its National Drug Code (NDC) and date of dispensing. Institutional review board approval was obtained at The University of Chicago and at United Healthcare.

### Cohort derivation

In our primary analyses, we identified patients with at least one colon cancer claim using the ICD-9-CM diagnosis code of colon cancer (153.x) in the top 3 diagnoses from January 1, 2007 to April 30, 2010. In order to restrict our cohort to incident cases, we used January 1, 2007 to June 30, 2007 as a 6-month “look-back or clean” period, and excluded individuals with a diagnosis of colon cancer prior to July 2007 [7]. We further excluded individual members with an insurance coverage gap greater than 6 months. In order to capture the full continuum of services and benefits provided, patients with coordination of benefits were also excluded. Additionally, a select set of members for whom UnitedHealthcare only provided administrative services were excluded because their data could not be used for research. Within the remaining cohort of patients with claims of colon cancer, we further excluded patients with claims indicating multiple primary malignancies. This criterion was applied to rule out patients with a malignancy other than colon cancer (e.g. lung cancer, breast cancer) who could potentially be treated with the same antineoplastic agents [8].

We used two methods to determine the metastatic colon cancer (mCC) cases. In our main analyses, we identified individuals as having mCC based on the receipt of at least one of four antineoplastic agents - irinotecan, bevacizumab, cetuximab and panitumumab - that are FDA-approved and recommended by available guidelines only for the metastatic disease setting. This method was chosen in order to avoid the inclusion of patients whose metastatic diagnosis represented a “rule-out” diagnosis (e.g. patients with Stage II colon cancer whose reason for a staging imaging study is “rule-out metastatic colon cancer” and are thus coded as metastatic patients) [9].

In a sensitivity analysis, we aimed to increase the positive predictive value and specificity of this method by decreasing the number of false positives (i.e. those labeled metastatic, when in fact they received one of these drugs in the adjuvant setting), by also requiring the presence of two diagnosis codes for metastatic disease (196.xx–199.xx) separated by 30 days or more [10,11]. As previously shown, requiring two metastatic codes to appear separated by at least 30 days, reduces the designation of metastases in initially non-metastatic patients to 5% [9]. Using this approach, we considered the metastatic codes 196.2x (intra-abdominal lymph nodes), 196.6 (pelvic nodes), 197.5x (large bowel) as locally advanced rather than metastatic disease [12]. Similarly, we omitted patients with the code 198.89 (secondary malignant neoplasm of unspecified site) because of its high false-positive rate [13].

### Definition of unsupported off-label use

We used the National Comprehensive Cancer Network Clinical Practice Guidelines in order to identify regimens that lack sufficient scientific support and thus that are not recommended in metastatic colon cancer [14]. These guidelines are developed by recognized clinical experts, and consist of recommendations based on clinical trial results, expert evaluation, outcome analyses, and clinical experience [15]. As such, they are regarded as reflecting the standard of care by health plans, large employers and other payers, including the Centers for Medicare and Medicaid Services [16]. We focused on three UOL regimens for which recommendations have not been changed in these guidelines during the study period: (1) use of single agent capecitabine after progression on one or more fluoropyrimidine-based regimens such as FOLFOX or FOLFIRI [17], addressed as "shown to be ineffective” in the NCCN guidelines; (2) use of bevacizumab beyond cancer progression on a prior bevacizumab therapy, addressed as "insufficient data to support"; and (3) panitumumab or cetuximab after cancer progression on a prior epidermal growth factor receptor (EGFR) monoclonal antibody, addressed as "no data, nor is there a compelling rationale". In 2012, a Phase III clinical trial of bevacizumab in patients with metastatic colorectal cancer whose disease had worsened following first-line treatment has further showed overall survival benefit with the addition of this drug [18,19]. This manuscript analyzed data from 2007 to 2010, prior to completion of this study, when evidence of its use was still scarce.

### Rules for defining therapies

We examined the daily drug use for each patient in order to identify these therapies. We defined the sequential progression of therapies based on their temporal relationships using the dates of initiation and discontinuation provided in the administrative claims. We considered a treatment as a next line of therapy when an addition or substitution of chemotherapy or biologic agent was observed and the resulting drug regimen lasted ≥ 28 days, the duration of at least one chemotherapy cycle [8].

Within the metastatic colon cancer claims, we identified patients with capecitabine claims. Single agent capecitabine was defined as its use not accompanied by any other chemotherapy drug both 14 days before and after capecitabine therapy. We were interested in patients who received single agent capecitabine after use of 5-fluorouracil or capecitabine in a prior line of therapy in the metastatic setting. Further, we identified patients who received bevacizumab in combination with either oxaliplatin or irinotecan. The switch of therapy regimens from bevacizumab and oxaliplatin to bevacizumab and irinotecan or vice versa was considered UOL use. Noteworthy, in order to better identify patients with disease progression on bevacizumab, we required that patients received a bevacizumab regimen for at least 28 days. By using this 28-day threshold, our goal was limit the number of patients included in the study who had their regimen changed due to acute toxicity rather than disease progression. The use of panitumumab after clinical failure on cetuximab, or cetuximab after failure on panitumumab, (ie, sequential use of an epidermal growth factor receptor [EGFR] inhibitor) was assessed by identifying claims that included both cetuximab and panitumumab. Observational evidence suggests that allergic reactions tend to occur within the first few weeks of administration of these agents when they do occur [20]. As such, in order to exclude those patients who were switched to the fully human monoclonal antibody panitumumab because of an allergic reaction to the murine chimeric monoclonal antibody cetuximab, only regimens that were administered for at least 2 months were included.

### Estimating costs of therapy

We estimated the costs of therapy acquisition using the average sales price (ASP) for the entire duration of UOL therapies, a statutorily defined price based on the national average of manufacturers' sales prices from two earlier quarters plus a 6% margin [21]. Medicare part B and most private payers use some form of ASP-based payment. We further considered prices of doses of bevacizumab at 5 mg/kg, panitumumab at 6 mg/kg and capecitabine at 1250 mg/m2 twice a day for 14 days for a 170 cm, 70 kg man [22].

## Results

### Cohort derivation

**Figure 1:**
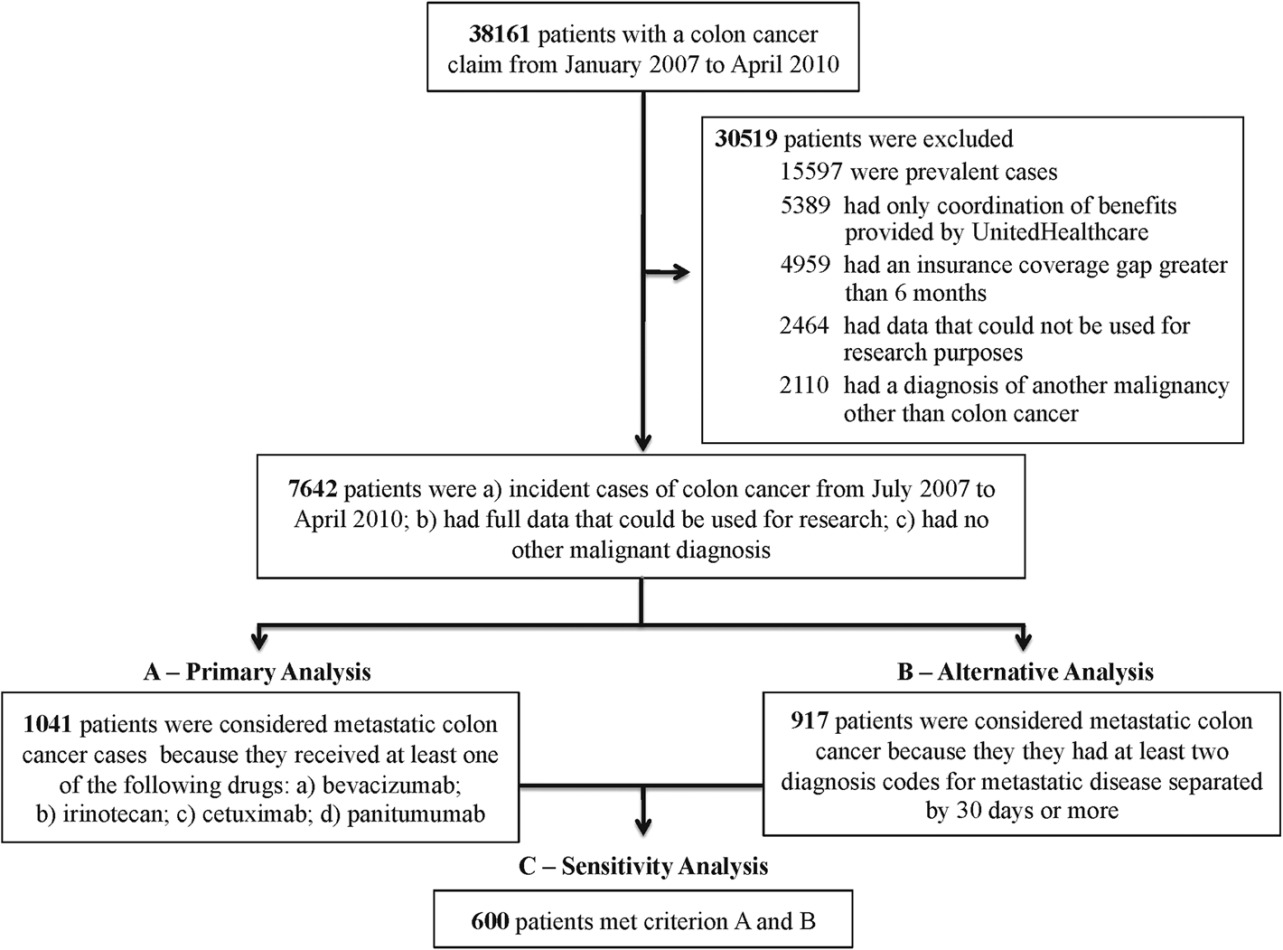
Algorithm for identifying patients with metastatic colon cancer.

From January 2007 through April 2010, we identified 38,161 patients with claims that included colon cancer. Of these, 22,564 cases (59%) were considered incident cases, with no colon cancer claims within 6 months prior to July 2007. We further excluded 4959 patients with a coverage gap greater than 6 months, 5389 coordination of benefit patients, 2464 patients whose data use was restricted by UnitedHealthcare’s administrative services only customers, and 2110 patients for having evidence of more than one malignancy. Our study cohort consisted of the remaining 7642 patients, reflecting those with full claims that included at least one first diagnosis code of colon cancer as the only malignancy from July 2007 to April 2010. See Figure 1 for a summary of the cohort derivation.

A total of 1041 (13.6%) patients received at least one of the four chosen agents to determine the presence of metastatic disease (irinotecan, cetuximab, panitumumab and bevacizumab). Age range and geographical data are listed in Table 1. In this privately insured population, 61% of patients were younger than 60 years.

**Table 1:** Age and geographic regions of patients with metastatic colon cancer in the primary analysis (N = 1041)

	**N (%)**		
	**All subjects 1041**	**Individuals receiving any of the three unsupported off-label therapies 139 (13%)**	***p*-value**
Age			
<50	277 (27)	44 (32)	0.014
50-59	358 (34)	52 (37)	
60-69	242 (23)	34 (24)	
>70	117 (11)	9 (6)	
Unknown	47 (5)	0	
Region			
Midwest	269 (26)	33 (24)	0.374
Northeast	107 (10)	17 (12)	
Southeast	370 (36)	41 (29)	
Southwest	126 (12)	21 (15)	
West	96 (9)	17 (12)	
Unknown	72 (7)	10 (7)	

### Use of UOL therapies

From the cohort consisting of 1041 patients, capecitabine was administered to 121 patients and 49 (40%) received it in an unsupported off-label context. Cetuximab was administered to 144 patients, while 38 patients received panitumumab. Notably, 6 patients (16%) received panitumumab after being on cetuximab at least 2 months, which was defined as UOL use. There were no patients who received cetuximab after being on panitumumab. Finally, bevacizumab was administered to 884 patients. Of those, 90 (10%) represented UOL use. In total, 139 (13.3%) individual patients received at least one of the three UOL regimens.

### Sensitivity analyses on cohort derivation to increase specificity

To further increase the specificity of our approach to identify metastatic cases, we identified 600 patients among those who had been considered metastatic cases based on administered drugs with at least two principal or secondary ICD-9-CM diagnosis codes indicating metastatic disease. In total, 116 (19.3%) of these 600 patients received at least one of these three UOL regimens, as shown in Table 2. Among these patients, 38 received UOL capecitabine; 79 patients received bevacizumab beyond progression, and 5 patients received panitumumab after progressing on cetuximab.

**Table 2:** Age and geographic regions of patients with metastatic colon cancer in the sensitivity analysis (N = 600)

	**N (%)**		
	**All subjects 600**	**Individuals receiving any of the three unsupported off-label therapies 116 (19%)**	***p*-value**
Age			
<50	163 (27)	37 (32)	0.153
50-59	233 (39)	46 (40)	
60-69	130 (22)	26 (22)	
>70	62 (10)	7 (6)	
Unknown	12 (2)	0	
Region			
Midwest	170 (28)	29 (25)	0.597
Northeast	63 (11)	13 (11)	
Southeast	194 (32)	35 (30)	
Southwest	68 (11)	17 (15)	
West	56 (9)	14 (12)	
Unknown	49 (8)	8 (7)	

### Number of claims and estimated treatment costs associated with UOL regimens in the primary analysis

There were 636 claims for bevacizumab beyond progression. Considering the costs of drug acquisition using the average sales price (ASP) for the entire duration of UOL therapies, we thus estimated that $1.34 million may have been used just to obtain that drug. Similarly, non-evidence based use of capecitabine was identified in 218 claims for a total cost of $718,000. And non-evidence-based use of panitumumab was identified in 19 claims at an estimated cost of $69,500. Within the 1041 metastatic colon cancer patients in the cohort, the estimated cost just to acquire the UOL drugs, which did not consider toxicities, ancillary support and infusion costs, was $2,127,500.

## Discussion

We found considerable utilization of expensive anticancer regimens in metastatic colon cancer without supporting evidence. We focused on treatments delivered after prior progression with the same or similar agents; more than one in eight patients received at least one regimen that is not considered standard of care. More importantly, the three assessed UOL uses have recommendations against their use in commonly adopted practice guidelines. With about 20,000 expected cases of colon cancer presenting with metastatic disease in 2011 [23], if we extrapolate an individual cost of $2043 per metastatic case, a total of $40 million may be related only to these 3 UOL regimens in new metastatic cases. In addition to unnecessary exposure to toxicities and the economic burden of these therapies, UOL regimens may undermine the ability to enroll patients into clinical trials to rigorously assess such UOL clinical applications. For example, a clinical trial assessing bevacizumab beyond progression in mCC, one of the therapies reported in this study, was closed due to poor accrual of patients in November 2010 [24].

Our findings are not the first regarding off-label use of prescription drugs. In a widely cited investigation, Radley et al [25] analyzed a nationally representative audit of office-based physicians and found that nearly one-fourth of all medication uses were for off-label purposes, and of these, nearly three-fourths lacked evidence of clinical efficacy. Despite this, less is known about the off-label use of oncology therapies, most of which are infusible drugs and not captured in office-based audits. In a prospective observational cohort study of previously untreated patients with metastatic colorectal cancer conducted to evaluate the safety and effectiveness of bevacizumab in combination with chemotherapy, approximately one-third of these patients received the non-evidence based bevacizumab beyond progression [26]. Similarly, an analysis of an integrated database of electronic medical records from 91 oncology practices indicated that among 1106 patients who received bevacizumab-containing regimens for metastatic colon cancer, 280 (25%) received it beyond progression in a subsequent line [8]. While this prior work considered all combinations of bevacizumab and other agents, we focused particularly on the use of bevacizumab and oxaliplatin after a prior use of bevacizumab and irinotecan and vice versa. To our knowledge, the UOL use of EGFR inhibitors and capecitabine has not been previously reported.

While our analysis was not designed for causal inference, there are several hypotheses regarding the UOL use that we document. First, patients with metastatic cancers and their treating physicians, when experiencing life-threatening illnesses with limited survival, may opt to receive and prescribe UOL regimens [27]. Many advocate that “even unapproved drugs often show early evidence of benefit reliable enough to use in desperately ill cancer patients” [28] or that “any glimmer of hope is better than none” [29]. Indeed, recent controversy surrounding the FDA’s decision to withdraw approval of bevacizumab for metastatic breast cancer highlights the degree to which advocacy may conflict with health policy [30]. Second, current reimbursement methods do not provide financial incentives for the delivery of many aspects of care that are promoted by professional guidelines. Notably, in the 2011 National Practice Benchmark drugs and infusion revenue accounted for 73% of total revenue in 37 oncology practices, while evaluation and management activities were responsible for only 8% of this revenue [31]. Third, mastering the clinical evidence can be a difficult task, and even compendia that attempt to do so may contain inaccuracies and fail to reflect timely standards of care [32]. Finally, the impact of marketing strategies on the UOL use of chemotherapy drugs is unknown [33]. Bevacizumab, panitumumab and capecitabine are all FDA-approved and ethically promoted drugs in the metastatic colon cancer setting. However, they are often administered without sufficient evidence, as was also the case of bevacizumab prior to 2012 [19].

Our report has strengths and limitations. We believe it represents the first effort to systematically quantify the utilization and estimated drug costs of non-evidence based off-label regimens in metastatic colon cancer in a large private insurance claims database. However, the use of claims is subject to misclassification bias and thus may include some patients without metastatic colon cancer and exclude some patients with metastatic colon cancer. In order to minimize the effect of such bias, we based our cohort derivation on previously published methods, and further increased the positive predictive value of the algorithm by including chemotherapy agents used in this cohort of patients. Nevertheless, coding imprecisions may still be present, such as the coding of panitumumab as J9999 (not otherwise classified antineoplastic drug) prior to the creation of its HCPCS J code in 2008. In addition, our analyses are limited to the privately insured and predominantly younger population, with limited number of older individuals. One might then hypothesize that physician aggressiveness may increase in younger patients, and thus the increased likelihood of treatment with UOL in this population. Also, as with all claims-based analyses, our data provide limited insight regarding specific reasons that regimens were selected or discontinued. This is a particularly important limitation when assessing bevacizumab beyond progression, when, for example, a regimen containing oxaliplatin may have been switched to irinotecan due to neurotoxicity rather than disease progression. Similarly, switching from a 5-fluorouracil based therapy (e.g. FOLFOX) to single agent capecitabine due to toxicity in a stop-and-go approach is an acceptable regimen, and it cannot be distinguished from salvage capecitabine after failure on 5-fluorouracil in claims analyses. In a prior analysis of patterns of care in metastatic colorectal cancer that included chart review for reasons for discontinuation, up to 18% of patients had their regimens changed due to issues related to toxicities and tolerability [34], and this should be seen as the main limitation of this study. Notably, in 2012 a Phase III clinical trial in patients with metastatic colorectal cancer whose disease had worsened following first-line treatment with bevacizumab plus standard chemotherapy (irinotecan or oxaliplatin-based) has showed overall survival benefit with the addition of this drug. This study analyzed data from 2007 to 2010, prior to evidence of this benefit, when the drug was still used as UOL [18,19]. Finally, our cost estimates are based on a payers’ perspective and limited to the therapies examined, rather than other potential costs of treatment such as treatment complications, lost wages, work productivity or emotional and quality of life.

## Conclusions

In view of the recent progress treating cancer, several newer therapies have been introduced at a rapid pace. In order to curtail costs in oncology while preserving efficacy and quality, a first step is identifying therapies with no or low value. This study provides us with hypothesis generating data that expensive UOL regimens are potentially being used in oncology, and further larger studies should further investigate causal relationships. In the policy arena, recent initiatives that incentivize providers to follow the best available evidence and reduce variations in care, such as the adoption of clinical pathways, as reported by Texas Oncology [35], or episode of care payments, piloted by UnitedHealthcare [36], hold the promise to decrease costs while improving outcomes, and should be further developed. Furthermore, discussions among several stakeholders about the appropriate use of practice guidelines, the level of evidence required for drug reimbursement, as well as the development of novel metrics to measure value in oncology become mandatory in our current health care debate. Utilization of unsupported and non-guideline based regimens in oncology, as well as factors associated with their use, may serve as targets for future health policy interventions.

## Competing interests

Dr. Newcomer and Monica Perkins own stocks and are employed by UnitedHealthcare. Dr. Ratain served as consultant for Genentech, received research funds from Bristol-Myers Squibb, and provided expert testimony for Mylan, none related to this work. There are no other known financial conflicts of interest among any of the authors including but not limited to employment/affiliation, all grants or funding, honoraria, paid consultancies, expert testimony, stock ownership or options, and patents filed, received or pending.

## Authors’ contributions

JADS, BP, MJR and GCA designed the study. MP and LNN were involved in acquisition of the dataset. JADS and GCA accessed the data and performed the statistical analysis. All authors helped with the interpretation of the data. JADS, BP, MJR, NJM and GCA drafted the first version of the manuscript, and all authors contributed to subsequent versions and revised it critically for important intellectual content. All authors read and approved the final manuscript.

## Pre-publication history

The pre-publication history for this paper can be accessed here:

http://www.biomedcentral.com/1472-6963/12/481/prepub
